# 阵发性睡眠性血红蛋白尿症诊断与治疗中国指南（2024年版）

**DOI:** 10.3760/cma.j.cn121090-20240624-00232

**Published:** 2024-08

**Authors:** 中华医学会血液学分会红细胞疾病（贫血）学组

## Abstract

阵发性睡眠性血红蛋白尿症（PNH）是一种罕见的后天获得性造血干细胞克隆性疾病，临床主要表现为血管内溶血、骨髓衰竭和高风险并发血栓等，虽为良性血管内溶血性疾病，严重者可危及生命。近年来，PNH在诊断及治疗等方面均取得较大进展，尤其是补体抑制剂治疗领域。为进一步规范和提高我国PNH的诊治水平，中华医学会血液学分会红细胞疾病（贫血）学组在广泛征集专家建议和意见的基础上，结合阵发性睡眠性血红蛋白尿症（PNH）最新诊治进展、国外相关指南/共识及我国国情对2013年版PNH专家共识进行更新，制订《阵发性睡眠性血红蛋白尿症诊断与治疗中国指南（2024年版）》。

## Abstract (en)

Paroxysmal nocturnal hemoglobinuria（PNH）is a rare acquired clonal disorder of hematopoietic stem cells characterized by intravascular hemolysis, bone marrow failure, and a high risk of thrombosis. Although PNH is a benign intravascular hemolytic disease, severe cases may be life-threatening. In recent years, remarkable progress has been made in the diagnosis and treatment of PNH, particularly in the field of complement inhibitor therapy. To further standardize and improve the diagnosis and treatment level of PNH in China, the Red Blood Cell Disease（Anemia）Group of the Chinese Society of Hematology updated the 2013 PNH Expert Consensus by combining the latest diagnosis and treatment progress of PNH, consulting relevant foreign guidelines/consensus and China's national conditions, and soliciting expert advice and opinions to formulate the Chinese Guidelines for the Diagnosis and Treatment of Paroxysmal Nocturnal Hemoglobinuria（2024）.

阵发性睡眠性血红蛋白尿症（PNH）是位于X染色体上的PIG-A基因突变导致的后天获得性造血干细胞克隆性疾病，其病理缺陷是糖基磷脂酰肌醇（glycosyl phosphatidyl inositol, GPI）合成异常而致由GPI锚连在血细胞膜上的一组锚连蛋白（CD55、CD59等）缺失，临床主要表现为血管内溶血（IVH）、骨髓衰竭和高风险并发血栓等。该病作为一种后天获得性溶血性疾病，没有先天发病的报道（先天性CD59缺乏除外），也没有家族聚集倾向。据统计PNH全球年发病率为1～2/100万人，患病率为10～20/100万[1]，而我国年发病率牡丹江地区曾统计为2.85/100万人[2]，属罕见病范畴。目前PNH已纳入我国《第一批罕见病目录》，因我国人口基数庞大，故PNH虽罕见但发病年龄在各年龄组均有报道，20～40岁约占77％。男女均可发病，男女之比为2.4∶1。

一、诊断与分型

（一）PNH克隆规范化命名：国际临床流式协会（ICCS）/欧洲临床细胞分析协会（ESCCA）指南规范了PNH克隆的命名，应用高敏感流式细胞仪检测，PNH细胞>1％称为PNH克隆，PNH细胞在0.1％～1％称为低量PNH克隆，PNH细胞<0.1％称为存在少量GPI缺陷细胞，或存在少量PNH表型细胞[3]。

（二）筛查PNH克隆的指征

1. 以血红蛋白尿、尿含铁血黄素阳性和（或）血清游离血红蛋白增高为主要表现的IVH。

2. 无法解释的溶血伴有铁缺乏、腹痛或食管痉挛、血栓栓塞、血小板减少和（或）白细胞减少。

3. 外周血成熟红细胞直接抗人球蛋白（Coombs试验）阴性、无明显肝脾肿大、极少见红细胞碎片、非感染性溶血性贫血。

4. 骨髓衰竭症：①怀疑或确诊的再生障碍性贫血（AA）或低增生性贫血；②难治性血细胞减少伴一系/多系发育异常；③不明原因的血细胞减少症。

5. 不同寻常的血栓形成：①年轻患者（尤其是45岁以下）；②非常见部位血栓形成：如肝静脉（Budd-Chiari综合征）、其他腹腔内静脉（门静脉、脾静脉等）、海绵窦、皮肤静脉等；③伴有下列征象/疾病的血栓形成：溶血征象、全血细胞减少、抗凝治疗效果欠佳、AA、骨髓增生异常综合征（MDS）。

6. 出现如下不能用其他疾病解释的临床表现：呼吸困难，腹痛、头痛、腰痛、吞咽困难、勃起功能障碍、体重减轻等。

（三）临床表现及症状分级

1. 临床表现：PNH发病隐匿，病程进展缓慢，常可累及全身多系统，出现较为复杂的临床表现。主要包括：①血红蛋白尿：典型的血红蛋白尿呈酱油或浓茶色，一般持续2～3 d，不加处理可自行消退，重者可持续1～2周，甚至更长时间。血红蛋白尿发作时可伴有发热、腰痛、腹痛等症状。②疲乏：90％以上的PNH患者出现疲劳，77％的患者为中度至重度。85％的患者主诉正受累于疲劳有关的痛苦中。③黄疸与肝脾肿大：半数PNH患者有轻度黄疸，约四分之一的PNH患者有轻度肝肿大，15％的患者有轻度脾肿大。④血栓形成：多以静脉血栓为主，少见动脉血栓。⑤肾功能不全：可表现为急性肾损伤（AKI）或慢性肾脏疾病（CKD）。⑥肺动脉高压（PHT）：50％的PNH患者出现PHT相关症状，其特征是肺动脉循环内压力升高。⑦全血细胞减少：许多PNH患者可以合并全血细胞减少、中性粒细胞比例减低。临床上表现为乏力、反复感染和出血倾向。⑧平滑肌功能障碍相关的表现：包括吞咽困难、呼吸困难、腹痛、胃胀、背痛、头痛、食管痉挛、勃起功能障碍等。

2. 贫血分级：极重度：HGB≤30 g/L；重度：HGB 31～60 g/L；中度：HGB 61～90 g/L；轻度：HGB>90 g/L。

3. 血红蛋白尿分级：频发：≤2个月发作1次；偶发：>2个月发作1次；不发：观察2年无发作（观察不足2年未发为暂不发）。

（四）实验室检查

1. 常规检测项目：

（1）血常规及凝血功能：RBC、HGB、网织红细胞百分比（Ret％）；WBC、PLT；成熟红细胞形态［有无异形和（或）红细胞碎片］、有无幼稚红细胞。血浆纤维蛋白原、D-二聚体水平等。

（2）多部位骨髓穿刺：双部位骨髓穿刺（不同平面）；骨髓涂片分析：造血组织增生程度；粒、红、淋巴细胞形态和不同阶段百分比；巨核细胞数目和形态；小粒造血细胞面积；是否有异常细胞等。

（3）骨髓活检：至少取2 cm骨髓组织（髂骨）标本用以评估骨髓增生程度、各系细胞比例、造血组织分布情况，以及是否存在骨髓浸润、骨髓纤维化等。

（4）肝功能：特别是血清胆红素（直接、间接）水平及血清乳酸脱氢酶（LDH）水平。

（5）肾功能、甲状腺功能、血清电解质以及病毒学检查［包括肝炎病毒、EB病毒（EBV）、巨细胞病毒（CMV）等］。

（6）血清叶酸和维生素B_12_水平。

（7）血清铁代谢指标水平：血清铁浓度、总铁结合力、转铁蛋白饱和度和血清铁蛋白水平。

（8）尿常规检查及尿含铁血黄素试验（实际检测的是脱落的肾小管上皮细胞中的含铁血黄素）。

（9）免疫球蛋白水平（IgG、IgA、IgM、C3、C4）及风湿免疫性疾病相关抗体。

（10）血清促红细胞生成素（EPO）水平。

（11）细胞遗传学：常规核型分析，必要时进行荧光原位杂交（FISH）以及遗传性疾病筛查（儿童或有家族史者推荐做染色体断裂试验）。

（12）血浆游离血红蛋白和结合珠蛋白水平。

（13）影像学检查：腹部B超、超声心动图、胸部CT及血管造影（如有肺动脉高压征象）、全身MRI检查（如有血栓栓塞征象）[4]等。

（14）流式细胞术检测外周血成熟红细胞和粒细胞CD55和CD59：流式细胞术检测GPI锚连蛋白缺失比例是诊断PNH最直接、最敏感的方法，高灵敏度流式细胞术已成为诊断PNH的“金标准”。白细胞是检测PNH克隆大小最精确的目标细胞；CD59作为红细胞上PNH检测的锚连蛋白，可鉴别正常细胞（Ⅰ型细胞）、部分缺失（Ⅱ型细胞）和完全缺失（Ⅲ型细胞）的PNH细胞。推荐使用CD235a-异硫氰酸素（FITC）、CD59-藻红蛋白（PE）用于高敏感检测[3],[5]–[7]。

（15）流式细胞术检测气单胞菌溶素前体变异体（FLAER）：FLAER是Alexa-488标记无活性的嗜水气单胞菌溶素前体的变异体，可特异性结合GPI锚蛋白，已成为检测PNH中性粒细胞和单核细胞高灵敏度流式细胞术分析的关键试剂。FLAER作用于所有GPI蛋白，不会因细胞表达GPI蛋白种类和多少的不同造成误差，因此是诊断PNH更敏感、特异的方法；FLAER检测微小PNH克隆更加敏感，且不受输血、溶血的影响。由于红细胞表面某些糖蛋白的存在使FLAER不能很好地与锚蛋白结合，因此FLAER一般只用于粒细胞和单核细胞的检测。粒细胞和单核细胞上的GPI锚蛋白主要选用CD24和CD14，CD157同时表达于单核细胞和中性粒细胞，可代替CD14和CD24。对粒细胞检测推荐采用FLAER和CD24/CD157，单核细胞采用FLAER和CD14/CD157。当FLAER和上述锚蛋白双阴性时确定为PNH克隆[6],[8]–[11]。

2. 可选检测项目：

（1）流式细胞术检测骨髓成熟粒细胞、有核红细胞、淋巴细胞、单核细胞CD55和CD59：如果PNH患者在检测前有多次输血史或存在重度溶血，外周血成熟红细胞PNH克隆检测可能受到输血和溶血的影响，而骨髓中的有核红细胞不受输血和溶血的影响，可避免漏诊。

（2）HLA高分辨配型：有异基因造血干细胞移植（allo-HSCT）指征的年轻患者建议与其兄弟姐妹行HLA高分辨配型，无同胞全相合供者可考虑亲缘单倍型供者、中华骨髓库或脐带血库寻找无关供者。

（五）诊断标准

1. 我国制定的PNH诊断标准：

（1）临床表现符合PNH。

（2）实验室检查：①Ham试验、糖水试验、蛇毒因子溶血试验、尿潜血（或尿含铁血黄素）等试验中有两项阳性。②流式细胞术检测发现：外周血中CD55或CD59阴性中性粒细胞或红细胞>10％（5％～10％为可疑），FLAER阴性中性粒细胞和（或）单核细胞>10％（5％～10％为可疑）。

临床表现符合，实验室检查具备①项和（或）②项者皆可诊断。建议有条件者完善流式细胞术检测以做精准诊断。

2. 国际PNH诊断标准：

（1）临床表现符合PNH。

（2）实验室检查：①流式细胞术检测发现外周血中CD55或CD59阴性中性粒细胞或红细胞>10％（5％～10％为可疑）。需要用流式细胞仪检测粒细胞、单核细胞或红细胞的2个不同细胞系中至少2种不同的GPI锚蛋白缺陷用于诊断，白细胞（粒细胞和单核细胞）比红细胞结果更可靠。②流式细胞术检测FLAER，分析白细胞GPI锚蛋白表达是否存在部分或完全缺失。

临床表现符合，实验室检查具备①项或②项者皆可诊断。

（六）鉴别诊断

PNH属于溶血性疾病，溶血性疾病根据病因及发病机制分为先天遗传性和后天获得性。在排除先天遗传性疾病后，PNH主要与后天获得性溶血性疾病相鉴别，需鉴别的主要疾病详见表1。

**表1:** 获得性溶血性疾病的鉴别诊断谱

获得性溶血性疾病	临床特征
自身免疫性溶血性贫血	病情程度变化颇大，自无明显溶血至严重致命性溶血不等，多数表现为乏力、头晕、体力活动后气短等贫血症状，分为温抗体型、冷抗体型及温冷双抗体型，Coombs试验主要检测血管内成熟红细胞上的自身抗体以证实温抗体型；冷凝集素试验用于检测血清中的冷凝集素以证实冷抗体型。
微血管病性溶血性贫血（HUS、TTP）	HUS多发生于儿童，发病前常有感染病史，该病主要累及肾脏，表现为少尿、高血压、严重肾损害等，神经系统症状较少见。血浆ADAMTS13活性常在正常范围。
	TTP多数伴有发热、精神症状、肾功能异常、凝血功能异常，与溶血组成“五联征”，ADAMTS13活性减低有助于遗传性TTP或发病初期的诊断。
遗传性多核幼红细胞伴阳性酸溶血试验	临床表现有贫血、轻度黄疸，肝、脾肿大。约半数红细胞脆性增高，形态不规则、嗜多色性，偶见有核红细胞。骨髓中幼红细胞增多，可见双核及多核幼红细胞。电镜检查可见部分幼红细胞有双层胞浆膜。Ham试验阳性。
感染所致溶血性贫血	病原体直接作用于红细胞的结果，常见致病菌包括产气夹膜杆菌、溶血性球菌、肺炎球菌、金黄色葡萄球菌、大肠杆菌等，临床特点为感染原发病的表现同时伴贫血、黄疸等。

**注** HUS：溶血尿毒综合征；TTP：血栓性血小板减少性紫癜

（七）临床分型

国际指南更新版[11]将PNH患者分为三种类型：（1）经典型PNH：存在PNH克隆，同时有溶血和（或）血栓的临床表现并除外其他骨髓衰竭性疾病；（2）PNH合并其他骨髓衰竭性疾病：主要是PNH合并AA/MDS/骨髓纤维化（MF）等骨髓衰竭性疾病；（3）亚临床型PNH：有少量或微量PNH克隆，但没有溶血或血栓形成的临床或实验室证据（表2）。我国PNH患者，经典型PNH占65.3％，合并骨髓衰竭性疾病占28.3％，亚临床型占6.4％，即我国PNH患者以经典型多见[12],[39]。

**表2:** 国际指南更新版临床分型[11]

分类	血管内溶血的速率	骨髓	流式细胞术
经典型	LDH显著增高伴阵发性肉眼可见血红蛋白尿	增生活跃伴红系造血旺盛或出现轻微形态异常	GPI-中性粒细胞>50%
合并其他骨髓衰竭性疾病	轻度（常伴溶血生化指标的轻度异常）	同时伴有骨髓衰竭证据（AA/MDS/MF）	GPI-中性粒细胞<50%
亚临床型	无血管内溶血的临床或生化证据	同时伴有骨髓衰竭证据（AA/MDS/MF）	使用高敏感的流式细胞检测手段可见<10%的GPI-中性粒细胞

**注** LDH：乳酸脱氢酶；GPI：糖基磷脂酰肌醇；AA：再生障碍性贫血；MDS：骨髓增生异常综合征；MF：骨髓纤维化

二、治疗建议

（一）总体治疗原则

PNH治疗方案的选择基于疾病临床分型，经典型PNH一线治疗为补体抑制剂，可选择依库珠单抗、可伐利单抗、B因子抑制剂。亚临床型PNH针对PNH克隆无需治疗，主要针对潜在骨髓衰竭进行治疗，应用免疫抑制剂可能有效。合并其他骨髓衰竭性疾病者建议应用免疫抑制剂联合促造血治疗，若患者PNH克隆比例较高且伴有溶血，可应用免疫抑制剂联合补体抑制剂；有合适供者的年轻患者可考虑行HSCT治疗（图1）[10]–[11],[13]–[20]。

**图1:**
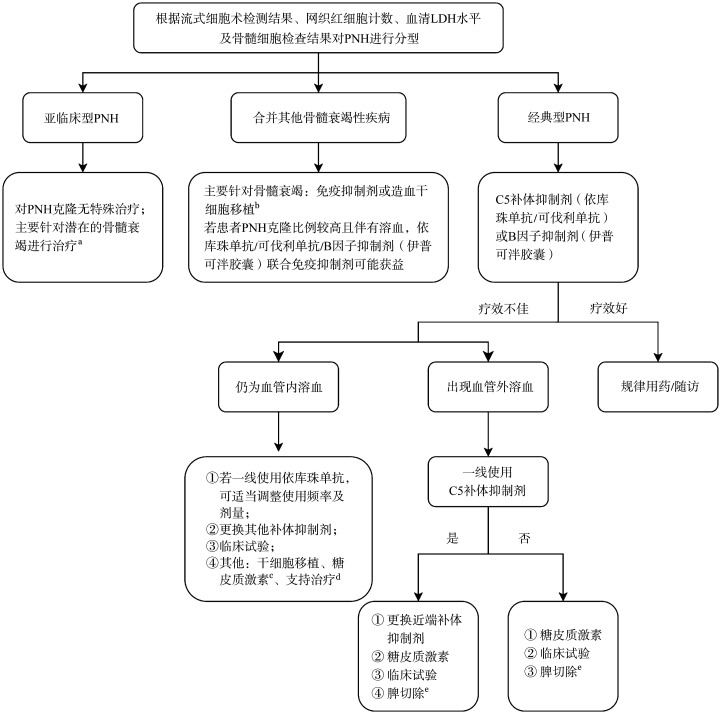
阵发性睡眠性血红蛋白尿症（PNH）治疗流程图 **注** ^a^ 部分研究提示患者对免疫抑制剂治疗效果好；^b^ 造血干细胞移植可消除PNH克隆，但免疫抑制治疗对PNH克隆大小无明显影响；^c^ 由于C3调理素沉积导致血管外溶血时可考虑应用；^d^ 包括成分血输注、促造血治疗；^e^ 脾大可尝试脾切除，同时应考虑血栓形成问题

（二）补体通路抑制剂治疗

1. 治疗指征及时机：补体通路抑制剂被批准用于粒细胞和（或）单核细胞PNH克隆>10％并伴有严重IVH，即LDH>1.5倍正常上限（ULN）的PNH患者，同时伴有终末器官损害的证据，包括：①贫血伴相关症状（伴或不伴输血依赖）；②与PNH相关的血栓形成（静脉或动脉）；③其他病理原因无法解释的肺动脉高压、肾功能不全、呼吸困难、腹痛等；④PNH合并妊娠患者[10]–[11],[13]–[14],[17]。

2. 补体抑制剂的选择及用法：

（1）重组人源型抗补体蛋白C5单克隆抗体：

①依库珠单抗：依库珠单抗通过减少慢性溶血，显著减少血栓事件，同时改善PNH患者的肾功能、呼吸困难，减少输血，实现疲劳减轻和健康相关的生活质量改善，最终延长PNH患者的寿命，实现生存获益。国际PNH登记研究（NCT01374360）（真实世界研究）显示，接受依库珠单抗治疗的患者与从未接受治疗的患者相比，20年的预估生存率分别为82％和69％，依库珠单抗治疗高疾病活动度（High disease activity, HDA）患者获益比非HDA患者更显著[22]。依库珠单抗于2023年11月12日被中国国家药品监督管理局（NMPA）批准治疗儿童及成人PNH患者，用药之始应充分告知患者永久性、持续性治疗的必要性。首先推荐的依库珠单抗维持剂量是固定的（每2周900 mg），而不是基于体重或体表面积。部分患者在14 d治疗周期结束时可能表现出突破性血管内溶血（BTH），即LDH升高和溶血症状的进展，这种情况下可通过将治疗周期缩短至13 d或12 d，或增加依库珠单抗的维持剂量改善[23]。依库珠单抗可以用于儿童、妊娠以及哺乳期患者。近端补体抑制剂使用中发生BTH，使用C5单抗等远端补体抑制剂挽救治疗。

②可伐利单抗：作为一种序贯单克隆抗体回收技术（SMART）抗体，可伐利单抗可快速持续控制IVH，维持血红蛋白稳定，降低输血需求，总体耐受性良好，治疗期间BTH发生率低[24]。该药由于其作用靶点不同于依库珠单抗，能克服补体C5多态性问题[25]。2024年2月NMPA批准可伐利单抗用于未接受过补体抑制剂治疗的成人和青少年（≥12岁）PNH患者。

③其他C5补体抑制剂：Ravulizumab（ALXN1210）作为新型、长效抗C5单克隆抗体，其作用靶点和依库珠单抗完全一样，不能克服补体C5多态性问题。于2018年12月被FDA批准用于未经依库珠单抗治疗的成人、青少年和儿童PNH（体重≥10 kg）患者和（或）高疾病活动度且经依库珠单抗治疗至少6个月后处于疾病稳定的PNH患者[26]–[27]，目前国内尚未上市。

（2）B因子抑制剂：

盐酸伊普可泮胶囊（Iptacopan）作为一种靶向B因子的近端补体抑制剂，可选择性抑制旁路途径，同时使凝集素途径和经典途径的直接信号传导保持完整，以控制末端补体介导的IVH及C3b介导的血管外溶血（EVH）[28]。于2023年12月被美国FDA批准治疗初治及对C5抑制剂疗效不佳的成人PNH患者；2024年4月被中国NMPA批准治疗未接受过补体抑制剂治疗的成人PNH患者，推荐剂量为200 mg，口服，每日2次，如果漏服1剂或多剂，建议患者应尽快补服1剂（即使与下一次计划给药时间很接近），然后恢复常规给药方案。

（3）C3补体抑制剂：

新型抗C5药物本质上不能满足C3介导的EVH的临床需求，针对C3或替代途径（AP）的新型口服抗补体抑制剂逐渐问世，对未经治疗的PNH患者或对补体C5抑制剂无效或仅部分应答的患者显示出良好效果[29]–[30]。Pegetacoplan可控制C5介导的IVH，并预防C3介导的EVH，于2021年5月被美国FDA批准治疗成人PNH患者及对C5抑制剂不耐受的PNH患者[31]。目前国内尚未上市。

（4）其他补体抑制剂：

其他C5补体抑制剂（Tesidolumab、Zilucoplan、Pozelimab、Coversin、CAN106等），C3补体抑制剂（ALXN-1102、APL-9、ARO-C3等），D因子抑制剂（Vemircopan、BCX9930）的相关临床试验正在开展中[32]–[35]。

3. 感染的预防及治疗：

（1）疫苗接种：补体抑制增加了脑膜炎球菌（奈瑟球菌）感染的风险，所有患者均须在接受补体抑制剂治疗之前至少2周进行疫苗接种，以降低感染风险，且每3年重复接种1次。疫苗接种未满2周因病情危重急需接受补体抑制剂治疗者，建议从补体抑制剂治疗开始至第2剂脑膜炎球菌疫苗接种后15 d采用抗生素治疗（青霉素或阿莫西林；红霉素或环丙沙星）预防脑膜炎球菌感染，单抗治疗本身无需终身抗生素预防，除非针对疑似感染的早期管理。建议PNH患者接种新型冠状病毒疫苗，即使接种后的炎症反应可能导致BTH，风险收益比仍建议接种。为了降低疫苗引发溶血的风险，疫苗接种时间应尽可能接近单抗治疗时间（补体抑制剂治疗后的第1周内），疫苗接种后第1周应密切监测临床相关症状，加强患者宣教[36]。

（2）突破性感染的治疗：如果PNH患者出现发热等症状，建议立即由医疗专业人员对其进行检查。如果怀疑脑膜炎球菌感染，建议立即使用第三代头孢菌素（非中性粒细胞减少型）或美罗培南（中性粒细胞减少型），同时应紧急联系为患者提供诊疗的PNH团队。当PNH患者出现突破性感染相关并发症时，不建议停止抗补体治疗[37]。

4. EVH的处理：C5补体抑制剂治疗期间，若出现持续贫血加重还需警惕红细胞C3沉积增加导致的EVH，此时需完善溶血相关指标、Coombs试验、腹部B超等检查进行鉴别诊断。出现补体C5抑制剂相关EVH，可尝试以下治疗方法：①近端补体抑制剂；②糖皮质激素；③临床试验；④脾大可尝试脾切除，同时应考虑血栓形成问题[22]–[23]。

（三）免疫抑制剂和促造血治疗

合并骨髓衰竭的患者可以给予环孢素A（CsA），甚至联合抗胸腺/淋巴细胞免疫球蛋白（ATG/ALG）治疗。同时，也可联合雄性激素（如司坦唑醇、丙酸睾酮、达那唑等）和粒细胞集落刺激因子、EPO等促造血治疗。

（四）allo-HSCT

1. 适应证：allo-HSCT是目前PNH的唯一潜在治愈方法。但PNH作为良性克隆性疾病，补体抑制剂已在国内上市使用，可使PNH患者取得与正常人群相当的生存期，对照较高的移植相关风险，补体抑制剂时代是否需要allo-HSCT治疗PNH，尚需依据患者的实际病情及各中心的移植经验充分评估风险及获益。补体抑制剂时代，PNH移植适应证包括：①补体抑制剂治疗失败；②PNH合并重型/难治型骨髓衰竭；③补体抑制剂不可及的严重经典型PNH；④PNH演变的MDS、急性髓系白血病患者。PNH allo-HSCT治疗后，建议每3个月进行1次随访，直至PNH克隆检测不出，以后每年检查溶血相关指标[10],[13]–[14]。

2. 供者的选择：以常规供者选择顺序（HLA相合同胞、HLA相合无关、单倍体相合）确定供者。

3. 预处理方案和移植物抗宿主病（GVHD）预防：同胞全相合移植以氟达拉滨（Flu）、环磷酰胺（Cy）联合ATG作为预处理方案，CsA预防GVHD。无关供者及单倍型移植以白消安（Bu）、Cy联合ATG作为预处理方案，使用CsA+霉酚酸酯（MMF）+短程甲氨蝶呤（MTX）预防GVHD[38]。到目前为止，国内外对于PNH预处理及GVHD预防方案尚无统一、明确推荐（尤其是替代供者移植），真实世界中各中心所用方案不尽相同。

（五）其他对症、支持及探索性治疗

1. 支持治疗：长期血红蛋白尿可导致缺铁，补铁治疗可使活性氧产生，易诱发血红蛋白尿，故确定缺铁后应从小剂量开始补铁治疗，为常规剂量的1/3～1/10，出现溶血加重者应立即停用。其他对症支持治疗包括红细胞、血小板输注及抗感染治疗。

2. 类固醇激素：肾上腺糖皮质激素可以保护PNH克隆、减少补体攻击和破坏，减轻溶血症状，缓解率达60％以上[39]。当发生急性溶血发作时，无可及补体抑制剂时可予肾上腺糖皮质激素（如泼尼松0.25～1 mg·kg^−1^·d^−1^，为避免长期应用的不良反应，应酌情短周期使用），辅以细胞膜稳定剂（维生素E）、叶酸、抗凝及碱性药物（如碳酸氢钠）治疗，对多数初诊患者能减轻溶血发作、稳定病情，但不建议糖皮质激素用于维持治疗[40]。

3. 减低剂量化疗：无补体抑制剂可及或其他原因导致无法使用补体抑制剂或allo-HSCT的情况下，针对反复发作的经典型PNH患者，溶血严重且支持治疗无效时，为有效地减少PNH异常克隆，最大限度地控制溶血，可采用减低剂量的DA（柔红霉素+阿糖胞苷）或HA（高三尖杉酯碱+阿糖胞苷）方案化疗，为避免出现化疗后骨髓抑制期的严重并发症（贫血、出血和严重感染），化疗采用的剂量宜偏小，疗程亦应缩短；应加强隔离和保护，预防感染；应重用造血因子以促进正常克隆恢复[41]–[42]。

（六）并发症的风险评估、治疗及管理

1. 并发症的风险评估：疾病本身严重程度的判断对于并发症的风险评估至关重要，欧洲药品管理局（European Medicine Agency, EMA）基于PNH患者血清LDH水平及临床症状定义了HDA：与溶血相关的LDH升高≥1.5 ULN，并且同时存在至少1种或多种相关症状：包括虚弱、疲劳、血红蛋白尿、腹痛、呼吸困难、贫血（HGB<100 g/L），主要血管事件（如血栓形成），吞咽困难或勃起功能障碍[43]。国际PNH登记研究（NCT01374360）显示每个HDA队列的患者百分比与血清LDH水平以及PNH克隆大小成正比，即血清LDH升高≥1.5 ULN、GPI缺失粒细胞克隆数越大，存在HDA可能性越大。相比于无HDA的PNH患者，HDA-PNH患者的血栓栓塞风险升高了2.07倍，且在LDH≥1.5 ULN的基础上，HDA标准症状越多，血栓栓塞风险越高，因此HDA患者尤其HDA症状多的患者预后更差[44]。

2. 并发症治疗及管理：

（1）血栓：

①预防：对于未应用补体抑制剂且粒细胞克隆>50％、D-二聚体升高、妊娠、围手术期状况和其他相关的血栓危险因素的患者，如果没有已知的抗凝禁忌证，可酌情应用低分子肝素或华法林进行一级预防。阿司匹林、氯吡格雷等抗血小板药物不能有效减少血栓形成风险，且PNH伴血小板减少患者易增加出血风险，不建议使用。在已经发生过与PNH相关的血栓栓塞事件的患者中，补体抑制剂可及时，建议使用补体抑制剂进行二级预防[45]–[46]。

②治疗：PNH合并血栓形成患者应酌情应用溶栓和取栓治疗，考虑PNH经常合并血小板减少，故血栓形成后，在抗凝、溶栓及出血之间需要权衡利弊。血栓形成的急性期用药首先考虑肝素或低分子量肝素（LMWH），后应用维生素K依赖性凝血因子拮抗剂。与普通肝素相比，LMWH生物利用度高，半衰期长，抗栓作用强、出血发生率低，故LMWH更合适。血浆制品含有高水平补体，由于补体阻断作用的丧失，会导致血栓形成和溶血的风险增加，故除紧急情况外，应用补体抑制剂的患者不应接受血浆制品，应用血浆制品的患者需立即追加补体抑制剂剂量。补体抑制剂可及时，应尽快应用以防止进一步血栓形成，如果血栓症状缓解，且患者在补体抑制剂的作用下溶血症状得到良好控制（LDH升高≤1.5 ULN），建议停止抗凝治疗[14],[16]–[17],[47]–[48]。

（2）肾损伤：PNH合并肾损伤是一种常被忽略或低估的临床特征，首先应提高临床医师对PNH患者肾损伤的认知，及时精确定义和准确判断肾损伤的分期，必要时肾科医师会诊、共同诊治。为了预防慢性肾衰竭（CRF），当补体抑制剂不可及时，应强调支持性措施，如补充铁或叶酸、定期输血和外源性EPO。急性肾损伤（AKI）的紧急处理包括液体复苏、支持性护理和必要时的血液透析；急性溶血发作时，需要紧急输血和给予糖皮质激素，采用水化、碱化、利尿等对症支持治疗，减轻肾前性因素对肾脏的损害，延缓肾功能恶化。同时行腹部超声及MRI检查排除肾静脉血栓形成。肾功能不全患者应用补体抑制剂是安全且耐受性良好的，尤其是在出现肾功能不全的早期即应开始应用[49]。

（3）合并妊娠：PNH患者妊娠期间，IVH发作更频繁，血栓栓塞并发症及母婴死亡率风险增加，依库珠单抗治疗前时期，PNH孕产妇和胎儿死亡率分别为8％～12％和4％～7％，因此所有妊娠的PNH患者都需要接受依库珠单抗治疗[50]。对于低疾病活动性患者，建议在妊娠前开始接受依库珠单抗治疗，持续治疗至妊娠结束及产后至少3个月，通过再次进行疾病风险评估做出是否可以终止治疗的决定。妊娠晚期和发生BTH的情况下，可考虑增加依库珠单抗的剂量。所有PNH粒细胞克隆超过10％的妊娠患者如没有抗凝治疗禁忌，建议在整个妊娠期间和产后3个月内使用LMWH进行治疗性抗凝治疗。妊娠期间有PNH克隆扩增的风险，如果目前没有接受依库珠单抗治疗，建议每2个月监测PNH克隆变化[47],[51]。即使依库珠单抗治疗可使PNH妊娠患者获益，该类患者仍需高度重视，强烈建议成立包括血液学家、妇产科专家及其他相关学科专家的多学科团队共同参与治疗和管理。

（七）儿童PNH治疗

1. 补体通路抑制剂：依库珠单抗和Ravulizumab均被FDA批准用于治疗儿童和青少年PNH，适应证为出现IVH性贫血、慢性疲劳、疼痛、呼吸困难，伴有或不伴有血栓形成的PNH患者。通过调整的基于体重的给药剂量已在儿童PNH患者中证实该方案有充分的补体抑制作用，而无药物累积[52]–[53]。脑膜炎球菌免疫方面，儿童PNH的研究比较少，目前尚没有脑膜炎球菌疾病发生的报道。鉴于成人PNH脑膜炎球菌感染的风险评估，建议所有儿童和青少年PNH患者在依库珠单抗和Ravulizumab治疗情况下，除了接种疫苗外，还要进行青霉素预防。此外，还需警惕淋病奈瑟菌感染，故需对患者进行安全性行为教育[54]。

2. allo-HSCT：儿童PNH患者allo-HSCT的应用取决于PNH的分型、是否有并发症以及骨髓衰竭的程度，推荐儿童PNH合并骨髓衰竭的患者可行allo-HSCT治疗。目前，关于儿童移植治疗的最佳时机、预处理方案的选择、供者选择以及补体通路抑制剂对移植建议的影响等问题尚未得到充分确定。

3. 免疫抑制治疗：针对儿童PNH合并骨髓衰竭的患者，适合移植但缺乏HLA相合供者，可选择糖皮质激素、环孢素A、雄激素和ATG治疗。在有经验的移植中心并得到家属充分理解知情的情况下可以考虑替代供者移植。

（八）疗效标准

1. 国内PNH疗效标准[55]：

（1）近期临床痊愈：1年无血红蛋白尿发作，不需输血，血常规（包括网织红细胞）恢复正常。

（2）近期临床缓解：1年无血红蛋白尿，不需输血，血红蛋白恢复正常。

（3）近期明显进步：按观察前后的病情分级，凡血红蛋白尿发作频度、贫血严重程度、骨髓增生状况中任何一项进步两级者为明显进步。

（4）近期进步：病情分级中任何一项检查有进步者。

（5）无效：病情无变化或有恶化。

如果观察期≥5年者可去除近期两字。判断治疗效果时须排除病情的自然波动。

2. 补体抑制剂治疗PNH疗效标准详见表3[17]。

**表3:** 阵发性睡眠性血红蛋白尿症（PNH）疗效标准

分类	输血频率	HGB水平	溶血指标和BTH发作
完全缓解	无	恢复正常^a^	LDH升高≤1.5 ULN、ARC≤150 000/µl且无BTH发生
显著缓解	无	恢复正常^a^	LDH升高>1.5 ULN和（或）ARC>150 000/µl，存在亚临床BTH
良好缓解	无	男性100 g/L≤HGB<130 g/L、女性100 g/L≤HGB<120 g/L	LDH和ARC均升高，存在亚临床BTH
部分缓解	无或偶尔（每6个月≤2次）	80 g/L≤HGB<100 g/L	–
微小缓解^b^	无或偶尔（每6个月≤2次）	HGB<80 g/L	–
	每6个月输血3~6次	HGB<100 g/L	–
	输血减少≥50%	HGB<100 g/L	–
无效^b^	每6个月输血>6次	HGB<100 g/L	–

**注** ARC：网织红细胞绝对值，ULN：正常范围上限，BTH：突破性溶血；–：不适用。^a^ HGB恢复正常指男性≥130 g/L、女性≥120 g/L；^b^ 对于不接受红细胞输注的患者，可以根据60 g/L≤HGB<80 g/L定义微小缓解，HGB<60 g/L定义无效

（九）随访

1. PNH患者应注重贫血、血栓、感染等相关症状的自我监测：例如疲劳、尿色改变、呼吸困难、疼痛、水肿、发热、勃起功能障碍等，有上述症状发生建议及时就医。PNH患者应用补体抑制剂治疗会增加感染的概率，需接种相关疫苗；一旦合并感染，应积极寻找感染灶和病原体，并给予针对性抗感染治疗。

2. 应用补体抑制剂治疗患者：应定期监测下列主要参数，包括：①血细胞分析、网织红细胞绝对值/百分比、血清LDH和胆红素指标：前3个月每月检测1次，后改为3个月1次。②肾功能、电解质和铁代谢指标（转铁蛋白饱和度、铁蛋白）：每3个月1次。③输血史信息：每6个月1次。④叶酸和维生素B_12_水平：如果存在叶酸/维生素B_12_缺乏的情况，建议每年检测1次。⑤PNH克隆比例：主要针对病情稳定的PNH患者，前两年每3～6个月1次，此后每年1次[17]。⑥补体活性：检测血浆中游离C5水平可用作末端补体活性的生物标志物；流式细胞术或Coombs试验检测C5抑制剂治疗后红细胞膜表面C3片段沉积可作为EVH评价指标。

3. allo-HSCT患者：建议移植后定期监测血细胞分析、溶血相关指标及PNH克隆变化，同时注意监测GVHD及感染症状；需动态复查骨髓穿刺（移植后14 d、28 d、42 d、2个月、3个月、6个月、12个月、24个月、36个月），包括形态学、DNA短串联重复序列（STR）、供受者性别不同者还需检测染色体及FISH等项目[15],[56]。

4. 其他确诊PNH患者：应常规监测溶血指标及PNH克隆变化。若病情稳定，可每年监测1次，同时监测是否有血栓并发症发生；出现任何临床或血液学参数变化时应缩短监测间隔，建议每月监测溶血指标及PNH克隆比例；亚临床型及合并其他骨髓衰竭性疾病患者，建议每3个月监测溶血指标、PNH克隆比例变化以及骨髓造血功能及免疫功能的恢复情况。
